# Towards Clinical Development of Scandium Radioisotope Complexes for Use in Nuclear Medicine: Encouraging Prospects with the Chelator 1,4,7,10-Tetraazacyclododecane-1,4,7,10-tetraacetic Acid (DOTA) and Its Analogues †

**DOI:** 10.3390/ijms25115954

**Published:** 2024-05-29

**Authors:** Ioannis Ioannidis, George Lefkaritis, Savvas N. Georgiades, Ioannis Pashalidis, George J. Kontoghiorghes

**Affiliations:** 1 Department of Chemistry, University of Cyprus, 2109 Nicosia, Cyprus; ioannides.c.ioannis@ucy.ac.cy (I.I.); lefkaritis.georgios@ucy.ac.cy (G.L.); georgiades.savvas@ucy.ac.cy (S.N.G.); paschalidis.ioannis@ucy.ac.cy (I.P.); 2 Postgraduate Research Institute of Science, Technology, Environment and Medicine, 3021 Limassol, Cyprus

**Keywords:** scandium radiopharmaceuticals, scandium theranostic pair, scandium radiolabeled complexes production and development, scandium imaging and diagnostic agents, chelating agents, DOTA, nuclear medicine

## Abstract

Scandium (Sc) isotopes have recently attracted significant attention in the search for new radionuclides with potential uses in personalized medicine, especially in the treatment of specific cancer patient categories. In particular, Sc-43 and Sc-44, as positron emitters with a satisfactory half-life (3.9 and 4.0 h, respectively), are ideal for cancer diagnosis via Positron Emission Tomography (PET). On the other hand, Sc-47, as an emitter of beta particles and low gamma radiation, may be used as a therapeutic radionuclide, which also allows Single-Photon Emission Computed Tomography (SPECT) imaging. As these scandium isotopes follow the same biological pathway and chemical reactivity, they appear to fit perfectly into the “theranostic pair” concept. A step-by-step description, initiating from the moment of scandium isotope production and leading up to their preclinical and clinical trial applications, is presented. Recent developments related to the nuclear reactions selected and employed to produce the radionuclides Sc-43, Sc-44, and Sc-47, the chemical processing of these isotopes and the main target recovery methods are also included. Furthermore, the radiolabeling of the leading chelator, 1,4,7,10-tetraazacyclododecane-1,4,7,10-tetraacetic acid (DOTA), and its structural analogues with scandium is also discussed and the advantages and disadvantages of scandium complexation are evaluated. Finally, a review of the preclinical studies and clinical trials involving scandium, as well as future challenges for its clinical uses and applications, are presented.

## 1. Introduction

Radionuclides, which are atoms with nuclei that undergo decay to form more stable daughter nuclei, have diverse applications in both the industrial and medical fields, playing a significant role in various aspects of modern life. Focusing on the medical applications of radionuclides, advances in medical technology have led to the development of new diagnostic and theranostic drugs, including cancer treatments based on the use of radiation/particle emission from radioactive substances [1,2]. Among such applications, within the domain of nuclear medicine, is the use of radiotherapies and radiopharmaceuticals, which have been proposed as extremely useful tools in aiding a variety of diagnoses and treatments, especially for cancer patients [1,2,3,4,5].

Radiotherapy is a method of treatment, mainly employed against cancer. High radiation doses are used to kill cancer cells and induce shrinking of tumors [4,5,6,7]. On the other hand, low radiation doses are used in the imaging of various parts of the body or physiological functions, such as imaging of organs, bones, metabolic pathways, and blood flow, in order to diagnose and treat various diseases [1,6]. There are two main types of radiotherapy, one based on using an external beam of radiation and the other based on applying internal radiation. The type of radiotherapy used in a patient depends on various factors, including the type of cancer, location and size of the tumor, as well as tumor proximity to normal tissues that are sensitive to radiation [1,3,8].

External beam radiotherapy, with ionizing radiation originating from a radiation source, is the most frequently utilized radiotherapy for cancer patients. In this case, the cancerous tumor and a limited area around it are treated by exposing the patient to high-energy X-ray radiation [1]. A different approach involves radiation emission from a radioactive source placed inside the body [1,8]. The radiation source can either be solid or liquid. Internal solid source radiotherapy is called brachytherapy and, like external beam radiotherapy, it is a local treatment targeting a specific part of the body. In the case of a liquid radiation source like chemotherapy, the treatment is systemic, reaching cells throughout the body by traveling through the bloodstream. However, unlike chemotherapy, these radioactive substances specifically target diseased cells, thus reducing potential side effects [1,3,9]. Both available treatments use radionuclide therapy, which is based on the administration of radioactive substances to patients and aims to stop the proliferation of cancer cells [1,2,6,10].

The use of radiopharmaceuticals and radiotherapies is an important tool, not only for the management of diseases and disorders, but also for the improvement of understanding of human diseases and for the development of new effective therapeutics [1,2,6,11]. In this context, there has been continuous, rapidly advancing progress in nuclear medicine, which is inextricably linked to the development of new methods of radiotherapy and to the efficient production of relevant radioisotopes, such as those of gallium (Ga) and scandium (Sc) [12].

The emphasis in this review is placed on scandium radioisotope chelator complexes. In particular, scandium has more than twenty radioisotopes, three of which are being studied for medical applications. Two of these are intended for imaging and diagnosis and one for therapeutic use.

A variety of factors affect the thermodynamic and kinetic stability of chelator radionuclide complexes and other metal complexes in vitro and in vivo [13,14,15]. Similarly, metabolic and other interactions, such as those with the plasma metal-chelating protein transferrin, could also influence the pharmacokinetics of radionuclide metal complexes, following their systemic administration [16,17,18]. In particular, stable radionuclide metal complexes, which do not readily exchange their metal with other chelators in physiological conditions, may have advantages for use in nuclear medicine.

Many chelators are potential candidates for stable scandium complex formation in vitro but only a few are effective in vivo. In particular, 1,4,7,10-tetraazacyclododecane-1,4,7,10-tetraacetic acid (DOTA) and some of its analogues appear to be a leading and promising group of chelators that form stable complexes with a variety of transition and rare earth metals, which, in turn, exhibit high stability under physiological conditions [19,20,21]. Most importantly, promising results from many in vitro and in vivo studies, as well as preliminary clinical findings, suggest that Sc-DOTA complexes are promising for further development in diagnostic and theranostic applications in cancer patients and nuclear medicine in general.

The aim of this review is to deliver a comprehensive account that encompasses developments in the area of scandium isotopes, spanning from production to clinical applications with DOTA and its analogues, along with an exploration of applied methodologies. The search methodology for the resources included prioritized recent research investigations, focusing primarily on the past two decades, and including recent developments with scandium and similar radionuclides in nuclear medicine. The review is intended to cater to the needs of investigators and educators as well as radiotherapy practitioners.

## 2. Methods for the Detection of Scandium and Other Radionuclides in Nuclear Medicine

Radiopharmaceuticals are a rapidly expanding sector of general pharmaceuticals, which are widely used in nuclear medicine. In particular, many radionuclides derived from different metals, including cobalt, chromium, technetium, and indium, are used in the form of metal-chelator complexes in radiopharmaceutical formulations [13]. Following administration of the radiopharmaceutical, the radionuclide/radioactive probe is traced in biological fluids or excretions or monitored in different parts of the body by specialized equipment, which has been developed for specific imaging related to clinical diagnosis or treatment. There is currently an increasing effort for the introduction and development of more advanced radiopharmaceuticals, such as scandium complexes with fine-tuned physicochemical and pharmacological properties, which enhances the prospect of further advancements in the fields of clinical diagnosis and therapy.

### 2.1. Overview of Radiopharmaceuticals and Radioimaging Methods

Radiopharmaceuticals are a category of pharmaceuticals that are widely used to diagnose and/or treat certain diseases. Examples include the treatment of certain types of cancer [22,23]. They can be administered to the patient via different routes and in various ways. For example, they can be taken orally, by intravenous injection, or directly placed at a target site organ, such as the eye or the bladder [1,22,24]. The radioactive tracers consist of a drug-carrier molecule conjugated to a radioactive atom and are used to diagnose various diseases. The choice of radioactive element and drug depends on the purpose of the scan. The types of specially designed cameras, that allow tracking the course of the radioactive probes, are Positron Emission Tomography (PET) scanners and Single-Photon Emission Computed Tomography (SPECT) scanners [25,26]. These two techniques are the most common imaging methods in nuclear medicine [1,3,25].

**Figure 1:**
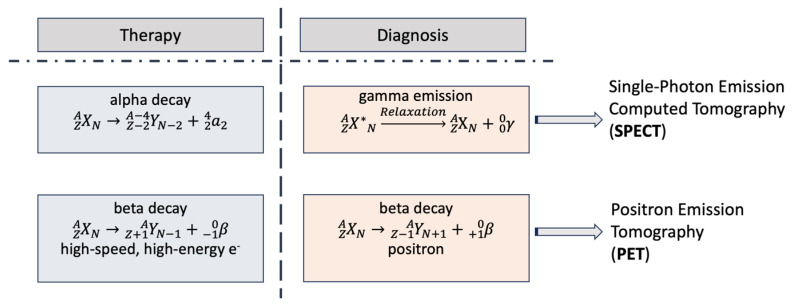
The differential properties of radionuclides used in nuclear medicine for either Positron Emission Tomography (PET) or Single-Photon Emission Computed Tomography (SPECT). Radionuclides that decay to alpha and beta (electron emission, e^−^) particles are used for radiotherapy, while those which undergo gamma emission and beta decay (positron emission, e^+^) are used for radiodiagnosis [1,29]. Abbreviations: PET: Positron Emission Tomography, SPECT: Single-Photon Emission Computed Tomography, A: Mass number, e^−^: Electron, N: Number of neutrons, X: Parent nucleus, X*: Excited nuclear state of parent nucleus, Y: Daughter nucleus, Z: Atomic number, α: Alpha particle, β: Beta particle, γ: Gamma radiation.

Some tracers use molecules that interact with a specific protein, carbohydrate, or even entire cells [1,27,28]. For example, in cases where physicians need to know the exact location of an intestinal bleeding, they can inject radionuclides into a sample of red blood cells taken from the patient [2,6]. The blood is then re-introduced into the patient’s body and a SPECT scan is used to determine the exact course that the blood follows in the body [6]. Any build-up of radioactivity in the bowel indicates the exact location of the bleeding problem [2,6]. The tracer used dictates whether a PET or SPECT scan is applicable, as shown in Figure 1 [1,29].

**Figure 2:**
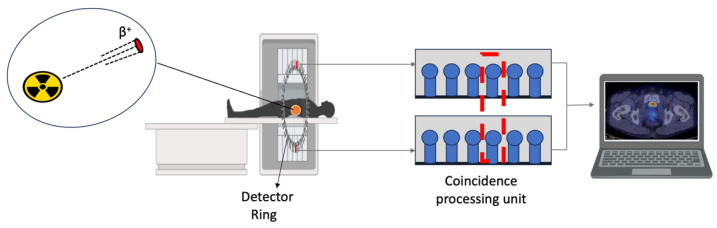
Positron Emission Tomography scan process. The two photons emitted at an angle of 180 degrees are detected at the detector ring. The pulse from the detector reaches the coincidence processing unit, which affords the position information. When an impulse is received simultaneously from two detector blocks, the detector electronics simultaneously monitor signals from each detector block and record counts (red dashed box). Abbreviation: β^+^: Positron.

Positron Emission Tomography (PET) measures cellular and tissue metabolic activity, useful in cancer detection and brain metabolism studies. For example, cancer imaging using a fluorodeoxyglucose (FDG) PET scan appears to be particularly useful for visualizing areas with increased glucose metabolism, thus helping to identify cancerous lesions. This method may be useful in cancer cells, which often have higher metabolic rates than normal ones. It could also be used for studying brain metabolism, thus helping in the diagnosis and monitoring of conditions like Alzheimer’s disease, epilepsy, and cancer [30,31]. It detects emitted photons from a radionuclide in the tissue, generated when a positron annihilates with an electron, producing two 511 keV photons [32]. These photons are detected in coincidence by a PET scanner, providing position information [33,34]. PET is valuable in brain and heart disease diagnosis, tumor localization, and visualizing biochemical changes in the body [1,30,31]. Radiopharmaceuticals are administered to aid visualization during the procedure [1]. It is noteworthy that in the past decade, there have been tremendous advancements in PET devices (Figure 2). The latest generation is a combination of PET and X-rays computer tomography (CT) or PET/CT, also known as digital PET, which is associated with the introduction of silicon photomultipliers (SiPM), a process that identifies the signal obtained from the emission of radioactivity and converts it to an image (Figure 2) [1,29,31].

The initial isotopes employed in PET were derived from elements naturally occurring in the human body, including O-15, N-13, and C-11. The main radionuclides used in radiopharmaceuticals for PET applications are F-18 and Ga-68. Furthermore, I-124, Y-86, Br-76, Cu-64 and Zr-89 are very common isotopes used in PET as they have demonstrated impressive efficacy in tagging monoclonal antibodies, while their half-lives align well with the pace of antibody accumulation in tumors or specific organs targeted for treatment. However, Sc-43 and Sc-44 are quite promising radionuclides for PET imaging, which are currently in preclinical studies for cancer diagnosis. Sc-44 is expected to be more advantageous, with broader applications and higher precision, in comparison to other radionuclides more routinely used in PET [29,35,36].

**Figure 3:**
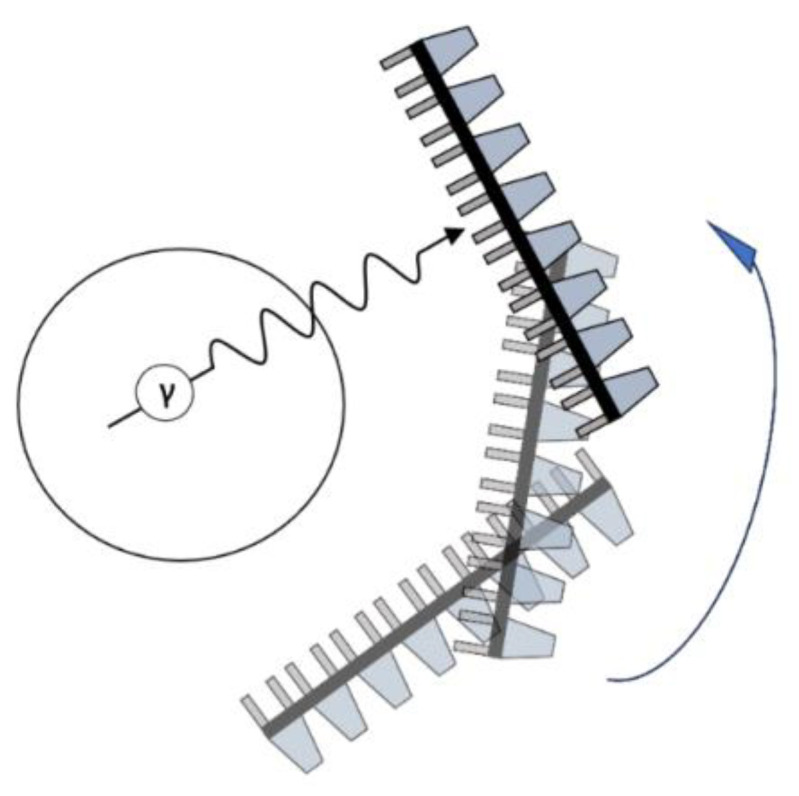
Detection of a photon by a Single-Photon Emission Computed Tomography scan gamma camera. The camera can detect only photons, which are at a right angle relative to the camera. The blue arrow shows the rotation direction of the camera. Abbreviation: γ: Gamma radiation.

Single-Photon Emission Computed Tomography (SPECT) is a three-dimensional and four-dimensional imaging technique that combines scintigraphy with computed tomography, offering higher resolution and accuracy compared to planar nuclear imaging [1,37]. It visualizes radionuclide distribution in three and four dimensions using γ-emitting radionuclides like Tc-99m and In-111 (Figure 3) [1,29,38]. SPECT data are obtained via a gamma camera, which detects radiation emitted by a radionuclide leaving the body [29,39]. The camera, with parallel holes, only accepts radiation emitted perpendicular to it by a thallium-doped sodium iodide crystal, measured by photomultipliers (Figure 3) [39]. Each incident photon yields three numbers: x and y values that indicate position in the scintillator, and energy (E). Successive angle views around the body generate a projection of the radiotracer distribution [39,40].

### 2.2. Radionuclides in Nuclear Medicine

The production of sufficient quantities and high quality of new radionuclides for use in nuclear medicine, either for treatment or diagnosis, requires systematic and extensive efforts from the stage of production to their clinical use [12,41,42]. Several key steps are crucial to ensuring a steady supply of radionuclides for medical applications, including cyclotron or nuclear reactor production, target material selection, isotope separation, radiopharmaceutical labeling, quality control, radiopharmacy distribution, clinical administration, regulatory compliance, radiation safety measures, patient-specific factors (e.g., health status and age), and diagnostic or therapeutic intent [12,41].

**Figure 4:**
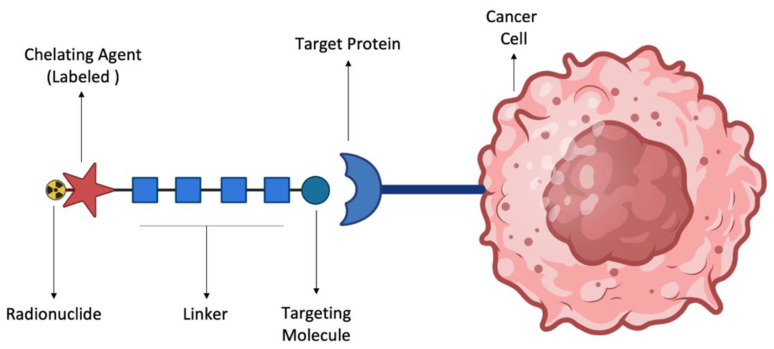
Graphical representation of radiopharmaceutical recognition by a target protein on a cancer cell surface. The graphical illustration shows a cancer cell with a mutant protein on its surface (e.g., an antigen). The radiopharmaceutical consists of the targeting molecule (e.g., antibody against the antigen) carrying a labeled chelating agent-radionuclide complex.

Identifying the ideal radionuclide to be employed in nuclear medicine, not only for imaging but also for therapy, is a very challenging task, as the appropriate choice is determined by several factors such as half-life, energy of emission, and radiation type. The radionuclide, in the form of a complex with a chelating molecule, must be recognized by the target to qualify for a particular application (Figure 4). The selection criteria for the appropriate radionuclide depend on its physical characteristics, such as half-life, particle emission, and gamma-ray emission, as well as production method, separation chemistry, and biological behavior [1,29,43].

For example, if an antibody with a relatively long biological time of action is employed, a radionuclide with an even longer half-life will be preferred [44]. The radionuclide must readily combine with the chelator, which is attached to the antibody, and the resulting complex should remain stable over the required period of treatment [45]. For imaging purposes, a shorter half-life is desirable as the patient must be exposed to radiation just for a limited time, but not so short as to limit its usability from production, delivery to the lab, administration time, and required imaging time [46]. It is also important for the radionuclide emission to reach the detector. The therapeutic radionuclide must emit photons that can be imaged, allowing both imaging and treatment under the lowest possible dose [29,47]. If gamma-ray photons are emitted during the decay process, the energy and the branching ratio should lie within the diagnostic range in which the dose received by the patient is minimized and the photon energy is less than 400 keV [48].

**Figure 5:**
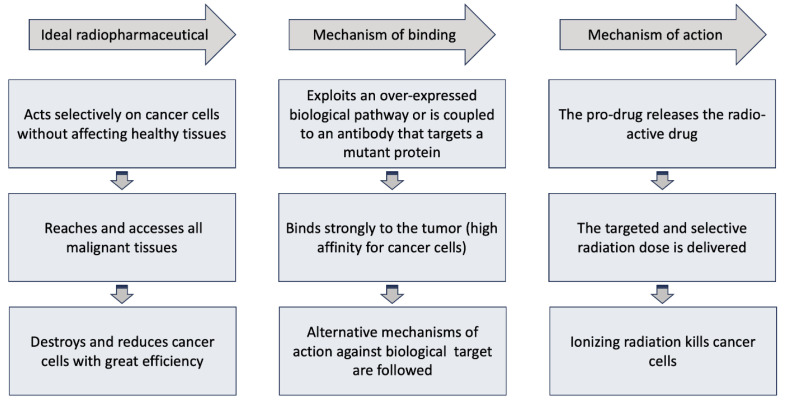
Schematic illustration of the properties, mechanism of binding and mechanism of action of an ideal radiopharmaceutical against cancer. The radiopharmaceutical should be able to emit sufficient radiation dose and destroy the cancer cells in a tumor without affecting normal cells in a healthy tissue.

The most successful radiopharmaceuticals used against cancer are those with high affinity for cancer cells and can strongly associate with the tumor but not with normal cells. There are several mechanisms of action against the drug target, as summarized in Figure 5 [48,49]. However, in the most common mechanisms, the pro-drug typically exploits an overexpressed biological pathway in cancer cells or is associated with an antibody that targets a mutant protein (antigen) on the surface of cancer cells, ultimately releasing the radioactive drug exclusively to these cells. Thus, these radioactive drugs selectively deliver targeted doses of radiation to tumors and their metastases, while avoiding healthy tissues [48]. The choice of the molecule that guides the radiation to the tumor is determined by its affinity for the target structures of the tumor, such as antigens or receptors [47,48,49]. The ionizing radiation emitted by radionuclides associated with the directing moiety-active drug conjugate, kills cancer cells, e.g., by damaging their DNA and disrupting useful proteins, through a cascading mechanism rooted in the ionization of water molecules inside the cell, eventually causing the shrinkage of cancerous tumors [49,50].

There are various other factors that need to be considered in choosing the ideal radionuclide, which can be summarized as follows: (a) Physicochemical properties: type of radiation, decay emission(s), energy of emission, and half-life; (b) The cost of the enriched target material and its recovery process, as well as its stability for long-term supply; (c) Access to available facilities for production and transport to the point of administration, within an acceptable time frame; (d) The cost of production and separation of the produced radionuclide, and (e) The simplicity and speed of the chemical separation method [41,47].

## 3. Properties and Effects of Scandium in the Context of Nuclear Medicine

Scandium is a xenobiotic soft metal, classified as a rare earth element. It is encountered in the +3 oxidation state (Sc(III)), which exhibits some similarities to Al(III). There are more than 20 scandium radioisotopes with variable half-lives, ranging from a few minutes to 84 days. There is an increasing interest for the use of the radioisotopes Sc-43, Sc-44, and Sc-47 in nuclear medicine.

### 3.1. The Role of Scandium for Imaging and Therapy in Nuclear Medicine

Metal radioisotopes play a pivotal role in the development of diagnostic and theranostic radiopharmaceuticals [51]. New diagnostic and theranostic drugs are being developed in nuclear medicine based on the use of radiation/particle emission of metal radioisotopes, including Sc-43, Sc-44, and Sc-47. In particular, the pursuit of radionuclide pairs that are ideally suited for use as a “theranostic or matched pair” has become a focal point in recent years, reflecting a growing interest in advancing the field of personalized medicine [51,52]. The concept of “theranostics” in nuclear medicine is based on the use of radionuclides, preferably isotopes of the same element, to enable the application of two identical radiopharmaceuticals with the same chemistry, pharmacodynamics, and pharmacokinetics, one for diagnosis and one for treatment [52,53,54]. A theranostic pair of radionuclides enables the simultaneous analysis of diagnostics tests and real-time monitoring of drug effects [54]. The uniformity of the two components in the “theranostic pair”, in this case two isotopes of scandium, both enhances the reliability of diagnostic tests and optimizes the efficacy of therapeutic intervention by providing a consistent, well-characterized platform [51,54].

There are various theranostic pairs that have been proposed in recent years beyond the isotopes of scandium, such as Cu-64/67, Sr-83/89, Y-86/90, I-124/131, Tb-152/161, and Tb-152/149. The first five pairs consist of a positron emitter combined with a β-emitting substance, while the last pair involves a positron emitter paired with an α-particle emitting substance. Some of these pairs have already been utilized in clinical research, while others are still undergoing development [55]. However, the positron emitter Sc-44 is currently attracting significant attention and has been clinically tested. Although the production methods for the therapeutic radionuclide Sc-47, which emits beta radiation, have been under investigation for over four decades, in recent years, with the emergence of the theranostic approach, there has been a notable increase in efforts dedicated to this pursuit.

**Figure 6:**
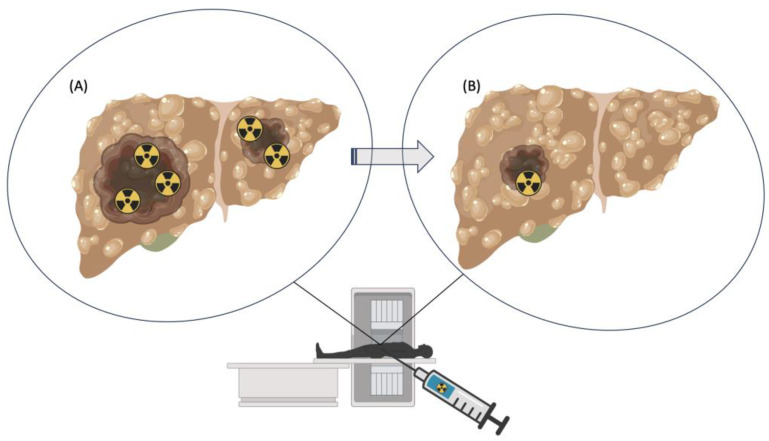
The effect of a theranostic pair in nuclear medicine, used for the simultaneous diagnosis and treatment of liver cancer. Cartoon images show liver tumors prior- (**A**) and post-treatment (**B**) with radioactive elements, in the form of a theranostic pair. The treatment results in elimination or shrinkage of tumors (**B**). The radioactivity symbol represents the radioisotopes used for radiotherapy and radiodiagnosis.

Scandium, a transition metal, exhibits a range of intriguing physical properties across its various isotopes. Primarily, its isotopes, ranging from Sc-43 to Sc-60, vary in atomic mass, which in turn affects their stability and reactivity, with Sc-45 being the only stable isotope. The density of scandium, approximately 2.985 grams per cubic centimeter, renders it relatively lightweight compared to many other transition metals [56]. Scandium is of particular interest for its potential applications in nuclear medicine, as stable scandium can be combined with F-18 and used as ‘pseudoradiometal’ [57] Moreover, scandium isotopes display notable magnetic properties, with some exhibiting paramagnetic behavior, while others demonstrate diamagnetic tendency. These diverse physical traits make scandium isotopes valuable in various applications, from alloying agents to research in nuclear medicine [55,56]. Scandium has three radioisotopes, which are being studied for medical applications: two for imaging and diagnosis and one for primarily therapeutic use [29,43]. Sc-43 and Sc-44 are quite promising candidates for PET imaging, with image quality comparable to the most commonly clinically used radionuclides, such as F-18 and Ga-68 [29,43,58,59]. Sc-47, on the other hand, emits β particles and is suitable for therapeutic purposes. It also produces γ-ray emissions that can be detected by SPECT imaging [29,58]. Therefore, Sc-43 or Sc-44 can be selected for diagnostics, whereas Sc-47 can be chosen for therapeutics, as shown in Figure 6 [60].

Positron Emission Tomography (PET) currently uses Ga-68, a radionuclide with a half-life of 68 min [61]. The application of Sc-43 and/or Sc-44, with half-lives of 3.9 and 4.0 h respectively, is a great advantage for PET [58,62]. The nearly four-fold half-life of Sc-43/Sc-44 would allow radiopharmaceuticals containing these isotopes to be shipped from their manufacturing facility to hospitals located far away. Additionally, with Sc-43/Sc-44, images could be recorded over longer periods of time [58]. Moreover, because scandium is stable when bound to 1,4,7,10-tetraazacyclododecane-1,4,7,10-tetraacetic acid (DOTA), combinations of radiopharmaceuticals can be used for both diagnostic and treatment options [63]. Sc-43/Sc-44 can, therefore, be used for diagnosis and, in combination with radionuclides Lu-177 and Y-90, to monitor targeted therapy, as Lu-177 and Y-90 are beta-emitting radionuclides, which release beta particles during radioactive decay. These particles have sufficient energy to penetrate tissues and can be used to deliver targeted radiation to specific cells, making them useful in targeted therapy for cancer. Moreover, Sc-43/Sc-44 can be combined with Ac-225, which provides an alpha emission detection for targeted alpha therapy [64]. However, several hurdles arise when working with Ac-225 due to the time-consuming and geometry-dependent nature of detecting alpha emissions. To mitigate this, gamma co-emission is employed, but it must be in equilibrium with the parent nuclide [65]. Scandium-47 is even more appealing, as it has the same kinetic characteristics with Sc-43 and Sc-44 for diagnostics and can be independently used in a medication derivative to treat cancer disorders [64,66,67]. However, Sc-47 cannot be currently manufactured in the volumes needed for therapeutic use [29,58].

### 3.2. The Production of Scandium Isotopes for Use in Medicine, Chemistry and Biochemistry

The production of new radionuclides for the purpose of producing new and innovative radiopharmaceuticals is a multidisciplinary collaboration between nuclear physicists, radiochemists, engineers, and radiopharmacists, among others [41]. The first stage in the process is the production, chemical separation, and quality control of the radionuclide.

**Figure 7:**
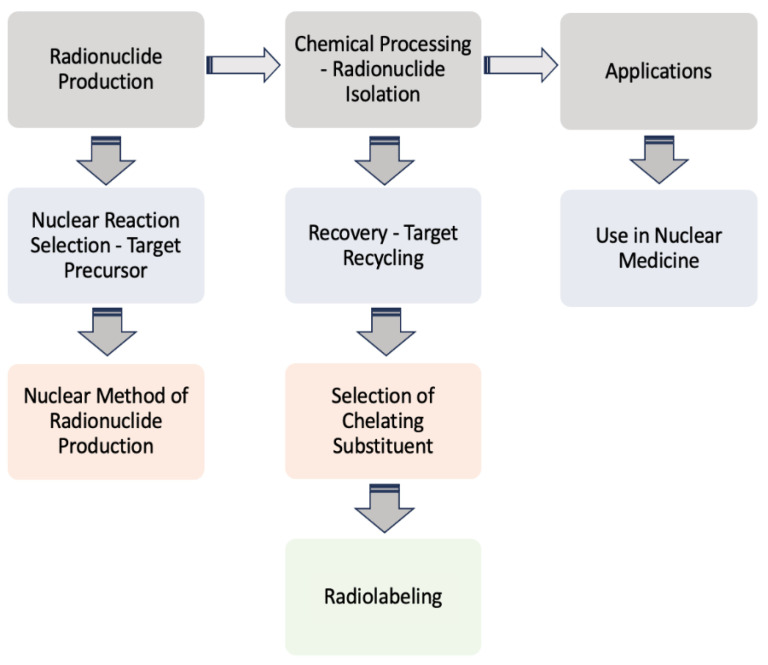
Summary of the chosen method or path from the production of the radionuclide to its use in nuclear medicine. The procedure involves initially the selection of an appropriate nuclear reaction (e.g., alpha, beta beams), the target precursor, the nuclear methods (e.g., cyclotron, generator), and, subsequently, the recovery/recycling of the target and the produced radionuclide’s isolation and conversion to a chelated complex, ready for application [29,53,64,68,69].

Scandium radioisotope production involves the generation and extraction of each of its radioactive isotopes [54]. Production typically begins with the selection of an appropriate target material, such as calcium or titanium, followed by irradiation of the precursor target using high-energy particles in a nuclear reactor or particle accelerator (Figure 7). Various nuclear reactions can be utilized depending on the chosen target material and desired scandium isotope. Once the target is irradiated, the resulting radioactive scandium isotopes are typically embedded in the target matrix. Subsequent chemical processing is required to extract the radioactive scandium isotopes from the target material. This extraction process often involves dissolution of the target material in an appropriate solvent, followed by separation of scandium from other elements present in the target matrix [60,68]. After extraction, the scandium isotopes undergo purification to remove impurities and contaminants. The purified scandium isotopes are then ready for further processing or radiolabeling to produce compounds for medical imaging and therapy applications. The purification process is crucial to ensure that the final radioisotope product is highly pure and suitable for its intended applications in nuclear medicine.

Scandium radioisotopes have diverse uses in different fields. In the medical field, for example, they can be employed in diagnostic imaging procedures, such as a PET scan [29,70]. In particular, the scandium radioisotopes Sc-43 and Sc-44 are typically incorporated into radiopharmaceuticals, allowing healthcare professionals to track the distribution of metabolites within the body. The process, involving the attachment of a radioactive isotope to a molecule, to generate a pro-drug, is essential for producing a form of the isotope able to enter the biological target system before being metabolized to the active drug [29,68].

The use of radiolabeled scandium isotopes extends beyond medical imaging [40]. In chemistry, these isotopes find applications in the study of reaction mechanisms, kinetics, and tracing experiments. By radiolabeling specific compounds or molecules with Sc-44 or Sc-47, scientists can gain valuable insights into the behavior of these species in chemical reactions, as well as their fate when the same reactions occur in biological systems.

### 3.3. Processes Applied for the Production, Isolation and Purification of Sc-43, Sc-44, and Sc-47

Many studies and efforts have been described for the preparation, isolation, and purification of Sc-43, Sc-44, and Sc-47. In this context, different approaches have been used for each radioactive isotope, involving specialised nuclear reactions and techniques.

Due to its low production rate/yield, high cost, and challenges in achieving sufficiently high isotopic purity, Sc-43 is currently more difficult to produce than other isotopes [29,59,67]. On the other hand, the exploitation of Sc-44 has recently progressed further in terms of production, preclinical research, and human application studies [29,43,61,67,68].

The sources used to produce radionuclides include nuclear research reactors, cyclotron facilities (emitting protons, deuterons, and alpha particles), and radionuclide generators, although the latter still require a reactor or cyclotron source to produce the parent radionuclide. The Ti-44/Sc-44g generator has been proposed by Roesch et al., and Filosofov et al. to produce Sc-44g [69,70]. Although the generator method has been extensively investigated, only a small number of facilities worldwide use these generators. Because of its long half-life and low cross-section (probability), the nuclear reaction ^45^Sc(p,2n)^44^Ti is used to manufacture the parent radionuclide titanium-44 (t_1/2_ = 60 y) [60]. Currently, one of the major challenges and drawbacks of producing Sc-44 using a generator is that the eluent requires post-elution purification to trace any Sc-44 radiolabeling reaction [71]. Purification is necessary due to the presence of competing chelating agents in the elution residue, such as oxalates derived from oxalic acid. The use of another column to remove the oxalates and isolate Sc-44 in a buffer suitable for radiolabeling could solve this problem. This process could be avoided if: (i) the eluate is free of competing chelators, (ii) the eluate is not too acidic, or (iii) the volume-to-activity ratio is low enough to maintain the total volumes of the radiolabeling reaction. A generator presented recently by Gajecki et al., used to isolate Sc-44 from the eluent, has met all the above-mentioned criteria [72]. This allowed the eluted Sc-44 to be used directly from the generator without post-elution treatment or loss of activity. Electronic linear accelerators are still under development [12].

Several studies have described nuclear reactions under different conditions for the production of Sc-43 and Sc-44 from calcium and titanium, with different outcomes in each case, in terms of the yields and purities of the various radionuclides produced [43,58,73,74,75,76,77,78,79,80,81,82,83,84,85,86,87,88]. These scandium isotopes can be produced via multiple routes. Studies have also been carried out to produce the isotopes in an accelerator using α-particles, protons, and deuterons from calcium and titanium nuclei (Table 1) [3,29,37,75,76,77,78]. Depending on the beam current and irradiation time, it has been demonstrated that the use of calcium and titanium can produce activities of the two isotopes in the order of GBq and hundreds of MBq [63]. However, the production of Sc-44 by these reactions is accompanied by the undesired co-production of its nuclear isomer, Sc-44m (t_1/2_ = 558.6 h). The same study also reports on production methods of Sc-43/44 isotopes.

Scandium-47 can be produced via multiple routes and its manufacturing has been extensively explored, particularly in recent years. Notably, its production in high yields and isotopic purity for use in pharmaceuticals has long posed a major challenge to researchers [29,72]. Production methods that have been thoroughly investigated include the use of cyclotrons with fast or thermal neutrons, use of protons, alpha rays, and, more recently, photonuclear reactions [77].

In this context, many studies have been reported for the nuclear reactions under different conditions for production of Sc-47, exhibiting different results in terms of yields and radionuclide purities (Table 2) [43,59,81,82,83,84,85,86,87,88,89,90,91,92,93,94,95,96,97,98,99,100,101,102,103]. In contrast to Sc-43 and Sc-44, the production of Sc-47 can proceed by using high-energy proton irradiation of titanium [104], calcium [105,106], and vanadium targets [100,107,108,109]. For example, in a recent method developed in CERN, Sc-44/44m and Sc-47 have been produced by irradiating thick target materials of ^nat^Ti, ^nat^V, and TiC pellets. Of all the target materials examined, ^nat^TiC stands out as the optimal choice for scandium mass separation using molecular halide beams, owing to its capacity for high operating temperatures and sustained release [110]. Despite the extensive studies on vanadium targets, the resulting yields of Sc-47 were relatively low in these cases. A few other methods were also reported, involving different nuclear reactions for the preparation of Sc-47, some with encouraging results [111,112,113,114].

In general, many separation methods have been reported in the literature for scandium isotopes and each of them is closely related to the type of the original target material/source, as described extensively elsewhere [70,71,72,73,74,75,76,77,78,79,80,81,82,83,84,85,86,87,88,89,90,91,92,93,94,95,96,97,98,99,100,101,102,103,104,105,106,107,108,109,110,111,112]. Similarly, different recovery yields, ranging from 45–98%, were reported for various systems, corresponding to the methods developed to allow separation of scandium isotopes from the target material [59,66,70,71,75,78,84,89,92,105,115,116,117,118,119,120,121,122,123]. Following the production of the scandium isotopes, the recovery of the calcium, titanium, and vanadium target materials is a very important process, owing both to their high cost and their potential for use as stable long-term supply sources [40]. The separation of the target source from scandium usually involves several processes of selective precipitation, filtration, and washing, aimed at maximizing extraction and purification [124,125,126,127,128] (Table 3). Different approaches have been used by various research teams in the course of these processes [129,130,131,132].

## 4. The Use of DOTA and Its Structural Analogues as Scandium Chelating Agents for Radiolabeling Purposes

Radiolabeling is often used in drug research and development, as well as nuclear medicine. It refers to the process of incorporating a radioactive isotope into a molecule or drug, to monitor its localization within the biological system of interest or to study its interactions with biological targets. Radiolabeling protocols require high incorporation yields, radiochemical purity, and molecular activity [133]. The labeling efficiency of the complexes used in radiolabeling applications is typically tested at variable pH values, temperatures, and metal-to-chelator molar ratios. It can also be monitored as a function of time to optimize the kinetics of the radiolabeled complex.

Metal radionuclide chelation is particularly important for both the isolation of radionuclides, which has been discussed in the previous paragraph, as well as their applicability in radiolabeling studies and nuclear medicine, since the resulting complexes need to be kinetically stable under the various conditions encountered during these applications [134].

Several chelating agents for scandium have been described in the literature in addition to the commonly used DOTA [135,136,137,138,139,140,141,142,143,144,145,146] (Table 4), many of which constitute derivatives or structural analogues of DOTA, with the same (DO3AP, DO3AP^PrA^, DO3AP^ABn^, DO3AM-NI, NBD), smaller (H_3_mpatcn, NOTA, AAZTA), or larger (TETA) macrocyclic ring system, while others are non-cyclic counterparts (EDTA, DTPA, H_4_pypa).

DOTA (entry 1, Table 4) and its analogues are of particular interest for inclusion in the design of radiolabeling applications, as they can form stable complexes with a variety of transition metals and rare earth metals with known radioactive isotopes. Importantly, these complexes exhibit high stability under physiological conditions, both in vitro and in vivo [147]. In the DOTA complex, scandium has a coordination number of 8, possessing a distorted square antiprismatic geometry. The Sc−N and Sc−O bond lengths in the complex are 2.44 Å and 2.15 Å, respectively [134]. The high stability constant of the [Sc(DOTA)]^−^ complex implies that in this rigid and highly symmetric structure, the DOTA chelator perfectly matches the coordination requirements of the metal cation [136]. An additional advantage of the combination of Sc(III) with DOTA chelator is that proton-mediated decomplexation in this case proceeds much slower when compared to the decomplexation of known lanthanide(III)-DOTA complexes that may also occur under the same conditions, thus rendering scandium potentially separable from lanthanides and preferable for radiolabeling applications [136].

It should be noted that several requirements must be met for the direct radiolabeling of DOTA with scandium(III), including acceptable acidity and temperature conditions, absence of competing chelators or metal ions, and efficient purification procedures [72,148]. Although DOTA forms very stable complexes, it exhibits rather slow formation kinetics at room temperature, with complex formation being facilitated by heating. Therefore, efficient radiolabeling of DOTA with scandium requires temperatures >70 °C [137], which remains a major drawback when radiolabeling DOTA-containing, heat-sensitive molecules, such as antibodies and oligopeptides [137,149].

The thermodynamic stability of several Sc(III) complexes with polyamino/polycarboxylic chelators belonging to the family of DOTA were determined in vitro by using the free-ion selective radiotracer extraction (FISRE), complemented by (cold-complex) ^45^Sc NMR and potentiometric titration data. The thermodynamic stability of the studied scandium complexes was found to follow the order: TETA < NOTA < EDTA < DTPA < DOTA [150]. The in vitro stability of these Sc(III) complexes was specifically assessed in the presence of hydroxyapatite (a mimic of bone composition) and rat serum to approximate physiological conditions. The two most stable complexes under these conditions, [Sc(DOTA)]^−^ and [Sc(DTPA)]^2−^, reached Sc-radioisotope incorporation of 90% and 80%, respectively. The suitability of DOTA as an efficient chelating agent for Sc(III) has also been confirmed in a more recent study [135].

In another recent study, the DOTA chelator was employed to titrate and quantify the produced ^43/44g^Sc, after it had been separated from the ^42/43/44^CaO source and trace metal contaminants (Fe, Zn, Cu). The resulting Sc-43 and Sc-44g complexes with DOTA were subsequently evaluated as radiotracers in PET/CT and performed comparably or even better than F-18, Ga-68, and Cu-64 radiotracers on the two clinical PET/CT scanners used [89].

The stability constants of scandium complexes of several analogues of DOTA bearing a pendant methylenephosphonic acid (DO3AP, entry 2, Table 4) or methylenephosphinic acid arm (DO3AP^PrA^ and DO3AP^ABn^, entries 3–4, Table 4) have also been investigated using potentiometry combined with ^45^Sc NMR spectroscopy [137]. A strong affinity of the phosphonic group for Sc(III) was suggested, with the DO3AP complexes being thermodynamically stable and kinetically inert, while exhibiting improved labeling efficiency at low temperature compared to DOTA. Furthermore, their complexes with scandium are also stable in vivo and show good pharmacokinetic properties, due to their high aqueous solubility. Additionally, no retention on bones was detected, a major concern due to the high affinity of phosphonates for hydroxyapatite component of bones; instead, fast excretion rates in urine favored these complexes. Nevertheless, these results also showed that the original [Sc(DOTA)]^−^ complex has higher in vivo stability compared to these analogues.

Further studies suggested that a 2-nitroimidazole-containing radiopharmaceutical complex Sc(DO3AM-NI) (entry 5, Table 4) forms relatively slowly in acidic solution and has a lower thermodynamic stability compared to the prototype compound, [Sc(DOTA)]^−^ [138]. Comparative studies indicated that the Sc(DO3AM-NI) complex exhibits superior inertness than the [Sc(DOTA)]^−^ and [Sc(AAZTA)]^−^ counterparts [9,105,127], rendering it a promising candidate for in vivo studies. It should be pointed out that complex inertness is essential for avoiding the occurrence of transmetallation and trans-chelation reactions taking place with competing endogenous metal ions and chelators, within the biological milieu [17,18].

The smaller macrocyclic chelating agent NOTA (entry 9, Table 4) has been considered somewhat inferior for scandium complexation because Sc^3+^ is not completely encapsulated within the cavity of the NOTA^3−^ macrocycle, which renders the complex Sc(NOTA) susceptible to de-metallation. However, this assumption has been disputed by X-ray studies, proposing stabilisation of the complex by a water molecule [143].

In an effort to address some of the aforementioned limitations of DOTA, new DOTA analogues have been designed for scandium to achieve room temperature complexation, such as the macrocyclic picolyl chelate H_3_mpatcn (entry 8, Table 4), the heptadentate AAZTA (entry 10, Table 4), and the non-cyclic chelator H_4_pypa (entry 13, Table 4). Despite the fact that the thermodynamic stability of [Sc(AAZTA)]^−^ was found to be lower than that of [Sc(DOTA)]^−^, a remarkable difference was observed in the radiochemical yield at 25 °C, indicating that AAZTA embedded Sc-44 more rapidly than DOTA [142].

Finally, certain challenges pertaining to the use of DOTA-like chelators still remain, especially the formation, in a non-selective manner, of competitive complexes with other transition metal cations, which, in some cases (e.g., Fe^3+^), are more stable than those for Sc^3+^, lanthanides, and Pb^2+^ [151,152]. Since various transition metal cations, such as iron, zinc, and copper may arise in the course of production of scandium isotopes from the target source, this lack of complexation selectivity creates an urgent need for a well-defined radiochemical process of purification and isolation of Sc-DOTA-type complexes to be developed.

## 5. In Vitro and In Vivo Preclinical Studies and Clinical Trials of Sc-44 and Sc-47

Drug development requires the collection of information from chemistry, biochemistry, physiology, pharmacology, and toxicology studies to enable drug optimization prior to clinical trials [153,154,155]. Similar information is necessary in studies developing drug conjugates, radiolabeled with scandium isotopes and intended for clinical use [156]. In these conjugates, the drug usually serves as a targeting moiety for directing the radiolabelled conjugate to the biological target, while the complexated radionuclide serves to either visualize the localization of the target or to induce therapy.

**Figure 8:**
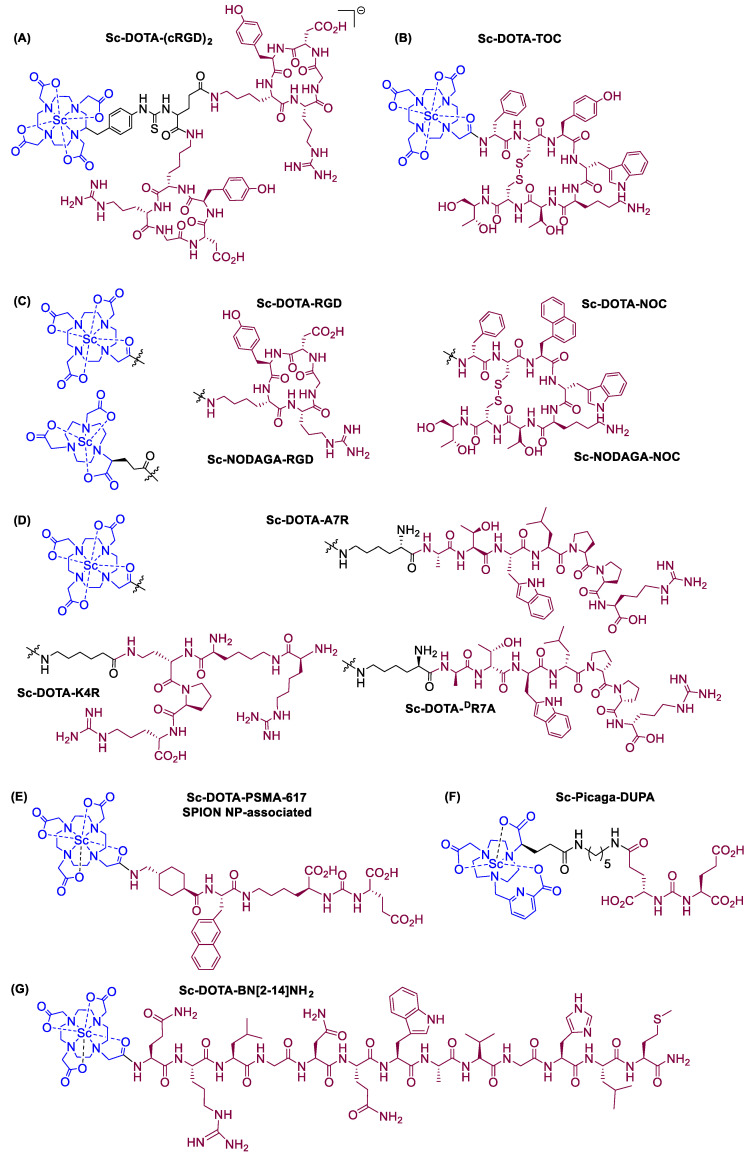
Representative examples (**A**–**G**) of Sc-radiolabeled conjugates between a metal chelator (blue) bound to scandium (Sc) and a targeting moiety (peptide or peptide-like small molecule, maroon). Some of these conjugates have been successfully used in vivo for delivering radionuclides to tumors, for imaging (Sc-44) or therapy (Sc-47) [63,140,141,156,159,160,162]. (Note: the chelator IUPAC names can be found in the “Abbreviations” section).

Many in vitro and in vivo preclinical studies have been carried out to investigate the effects of Sc-labeled DOTA (or DOTA analogues) covalently linked to a peptide (the targeting moiety) on various targets of anticancer interest [43,157,158]. Representative examples are compiled in Table 5 and the structures of such conjugates are shown in Figure 8.

**Table 5:** Summary of representative in vitro, ex vivo and in vivo studies, as well as clinical trials of Sc-44 and Sc-47 complexes with DOTA analogues or DOTA-containing conjugates.

Entry	Type of System	Isotope	Complexating Agent	Targeting Moiety in Conjugate	Biological Target	Detection Method	Reference
**1**	In vitro, in vivo, ex vivo	Sc-44	DOTA	cRGD peptides	integrin α_v_β_3_	PET	[156]
**2**	First in-human trial	Sc-44	DOTA	TOC peptide	liver and lymph node metastases	whole-body PET/CT	[159]
**3**	In vitro, in vivo	Sc-44	DOTA or NODAGA	RGD or NOC peptide	U87MG and AR42J tumors	PET/CT	[160]
**4**	In vitro	Sc-44	DOTA	A7R, ^D^R7A or K4R peptide	neuropilin-1 (NRP-1)	ELISA	[63]
**5**	In vitro, in vivo	Sc-44, Sc-47	DOTA	PSMA-617/ SPION NP	prostate-specific membrane antigen (PSMA)	PET/MRI	[140]
**6**	In vitro, in vivo	Sc-44 Sc-47	H_3_mpatcn/picaga	DUPA	PSMA	PET	[141,161]
**7**	In vitro, ex vivo, in vivo	Sc-44, Sc-45	DOTA	BN[2-14]NH_2_	GRPR	PET	[162]
**8**	In vitro, ex vivo, in vivo	Sc-44	DO3AM	2-nitroimidazole	hypoxia tumor	PET	[138]

Abbreviations: A7R: Ala-Thr-Trp-Leu-Pro-Pro-Arg, BN: Bombesin, cRGD: Cyclic arginylglycylaspartic acid, CT: Computed Tomography, ^D^R7A: DArg-DPro-DPro-DLeu-DTrp-DThr-DAla, ELISA: Enzyme-Linked Immunosorbent Assay, GRPR: Gastrin Releasing Peptide Receptor, K4R: Lys(hArg)-Dab-Pro-Arg, MRI: Magnetic Resonance Imaging, NRP-1: Neuropilin-1, PET: Positron Emission Tomography, PSMA: Prostate-Specific Membrane Antigen, SPION NP: Super-Paramagnetic Iron Oxide Nanoparticles. (Note: The IUPAC names of all chelators included in this table can be found in the “Abbreviations” section).

For example, one study targeting the protein α_v_β_3_, an integrin linked to tumor aggressiveness and metastasis in many cancer cell lines, involved in vitro, in vivo, and ex vivo evaluation of a radiolabeled conjugate (entry 1, Table 5) [154]. The conjugate, ^44^Sc-DOTA-(cRGD)_2_ (Figure 8A), resulted from ligation to Sc-44-labeled DOTA of two RGD-containing cyclic peptides, which are known binding partners of α_v_β_3_. The in vivo studies were carried out in female mice bearing U87MG human glioblastoma xenografts, and a PET scan was used to assess tumor targeting ability, specificity, and binding potential of the conjugate. Following the administration of the radiotracer to the mice, the interaction of the conjugate with the integrin α_v_β_3_ took place, thus allowing the integrin to be (non-invasively) visualized and quantified, showing increased accumulation in the tumor. An accumulation was also evident in the bladder. The study concluded that the integrin target is overexpressed in cancer and vascular cells. The ^44^Sc-DOTA-(cRGD)_2_ conjugate biodistribution in the integrin-rich tumors was found to be (3.93 ± 1.19), (3.07 ± 1.17), and (3.0 ± 1.25)% ID/g at 0.5, 2, and 4 h after administration, respectively. A competitive experiment with additional free cRGD showed inhibition of tumor labeling by the scandium conjugate, suggesting that this conjugate associates with the same integrin target as the well-characterized cRGD peptide.

A study investigating the potential of Sc-44 for labeling DOTA-conjugated peptides or other targeting vectors revealed a high labeling efficiency (>98%) with DOTA-D-Phe^1^-Tyr^3^-octreotide (TOC), another tumor-targeting cyclic peptide [143]. Following this promising result, the first in-human trial of ^44^Sc-DOTA-TOC (Figure 8B) was carried out on four cancer patients (ages 44–70) with metastatic neuroendocrine tumors (entry 2, Table 5), with imaging being performed at various intervals, from 10 min to 24 h post-administration, by means of a whole-body PET/CT scan [159]. This tracer exhibited a highly specific uptake in liver and lymph node metastases, both in patients with progressive disease as well as patients with stable disease, even allowing for the detection of a new metastasis. It was deemed to be a highly sensitive tracer, safe for human use, and at least as efficient as the previously known ^68^Ga-DOTA-TOC. It is suitable for late imaging (up to 24 h post-administration), thus overcoming limitations of previous PET tracers with shorter half-life radioisotopes [159]. This conjugate was deemed by the authors to be ideal for use with a ^44^Sc/^47^Sc theranostic pair.

Combinations of two metal chelators, DOTA and NODAGA, with two tumor-targeting cyclic peptides, RGD and NOC, led to the formation of four covalent conjugates, which were then radiolabeled separately with Sc-44 or Ga-68 in order to compare the performance of Sc-tracers (Figure 8C) with their Ga-counterparts, in vitro and in vivo [160] (entry 3, Table 5). The DOTA-based conjugates were readily labeled with both Sc-44 and Ga-68 and remained stable for more than four half-lives. In contrast, the radiolabeling of the corresponding NODAGA-based conjugates with Sc-44 proved more challenging, while these tracers also appeared more susceptible to metal challenge (i.e., metal exchange due to presence of another cation, Fe^3+^ or Cu^2+^) in vitro. However, in vivo, in mice bearing U87MG and AR42J tumor xenografts, the ^44^Sc-NODAGA-peptides showed similar tissue distribution to tumors as the ^44^Sc-DOTA-peptides, with no signs of instability. Biodistribution of the Sc-44-labeled conjugates, as judged by PET/CT imaging, was comparable to that of the corresponding Ga-68 conjugates in various tissues, with a marked superiority of Ga-68 only in liver tissue. The possibility of using the more reliable DOTA-based carriers with both Sc-44 and Sc-47 in the form of a theranostic pair was suggested by the authors [160].

A series of three ^44^Sc-DOTA-type radiotracer candidates with different linear or branched peptides (A7R, the retro-reverse ^D^R7A, and K4R) as the targeting moieties (Figure 8D) were tested for site-specific action on neuropilin-1 (NRP-1) co-receptor (entry 4, Table 5), a key component of a pro-angiogenic protein complex involving VEGF-A_165_, responsible for inducing an angiogenesis signaling pathway in tumors [63]. Apart from serving as imaging agents for NPR-1, the peptides were also designed to inhibit the angiogenesis pathway promoted by this complex. In vitro, two of the tracers (^44^Sc-DOTA-A7R and ^44^Sc-DOTA-K4R) showed high affinity for NRP-1 based on the results of a competitive ELISA assay, in contrast to their affinity for the retro-reverse ^44^Sc-DOTA-^D^R7A, which, unsurprisingly, showed no affinity, since all amino-acids in its structure had opposite stereoconfiguration compared to the ones in the A7R peptide. They also showed hydrophilic behavior, as well as considerable resistance against ligand exchange challenges imposed by cysteine and histidine. However, these peptides exhibited unsatisfactory nano-scale stability in human serum and were ultimately deemed unsuitable for specific use as therapeutic radiotracers.

A multimodal radiopharmaceutical conjugate prototype for the combined prostate cancer diagnosis and treatment was developed by employing a superparamagnetic iron oxide nanoparticle (SPION) platform (Figure 8E and entry 5, Table 5) [141]. The SPION was associated with the radiolabeled conjugate, while the magnetic properties of the SPION rendered the entire construct suitable for detection via MRI. The targeting moiety in this case was a small molecule (PSMA-617), which was covalently bound to the chelator DOTA. The intended target was the prostate-specific membrane antigen (PSMA) peptide, overexpressed in all forms of prostate tissue and exposed on the cell’s extracellular surface, for which PSMA-617 has high binding affinity. Both Sc-44 and Sc-47 were used as radionuclides, in the form of a theranostic pair, to afford diagnostic (detected via PET) and pharmaceutical function, respectively. The radiolabeling efficiency with scandium(III) was found to be greater than 97%. In this case, the combination of the β-radiation resulting from the decay of Sc-47 with SPION oscillation-induced magnetic hyperthermia (T > 42 °C) appeared to act against prostate cancer cells. The radiolabeled bio-conjugate showed higher affinity and cytotoxicity towards the human prostate cancer cell line LNCaP (PSMA^+^) compared to those for PC-3 (PSMA^−^) cells. It was also suggested that the magnetic properties of this bio-conjugate should allow its use in gradient-guided magnetic field drug delivery, since an externally applied magnetic field helps to both guide and retain the SPION construct localized at the area of the tumor. The uptake of the nanoparticles in a prostate tumor model in mice was positively confirmed by MRI.

The prostate-specific membrane antigen (PSMA) provided a target for yet another radiolabeled tracer, termed ^44^Sc-pigaca-DUPA [142] (Figure 8F and entry 6, Table 5). This comprises the superior chelating ligand H_3_mpatcn/picaga, allowing it to benefit from room-temperature formation of the Sc-complex, thus by-passing the requirement for submitting the peptide-like conjugate to high temperatures, as would be required in DOTA cases. The chelator is attached to a polycarboxylate-urea fragment (DUPA), similar to the one employed in the previous example. This stable conjugate was used to image, via PET, PSMA-expressing tumors in a pre-clinical mouse model. The same team reported the use of a similar conjugate with Sc-47, along with the use of other radionuclide tracers, to induce shrinkage of prostate tumors in mice with PSMA-expressing xenografts [161].

A conjugate that combines the DOTA chelator with a bombesin peptide analogue (BN[2-14]NH_2_) was labeled with either Sc-44 or natural isotope Sc-45 as a “cold-complex” mimic (Figure 8G and entry 7, Table 5) [162]. Bombesin targets the gastrin releasing peptide receptors (GRPR), which are expressed in prostate cancer cells. The ability of the natural scandium complex to bind the GRPR was assessed in vitro by means of a displacement assay on PC-3 cells in the presence of competitive radiolabeled ^125^I-Tyr^4^-BN. This indicated that ^nat^Sc-DOTA-BN[2-14]NH_2_ is an efficient binder, albeit one order of magnitude weaker than the respective ^nat^Ga “cold-complex”. In vitro internalization/efflux studies on PC-3 cells with the Sc-44-radiotracer indicated specific uptake, which was faster (91.9 ± 10.1% in 30 min) compared to the Ga-68 counterpart (83.0 ± 3.4% in 60 min). The Sc-complex was also found to be retained for a longer period than the Ga-complex. Ex vivo organ distribution experiments in rats showed accumulation of the scandium radiotracer in the pancreas, which can be attributed to the expression of pancreatic GRPR. In vivo PET imaging in male Copenhagen rats bearing the R-3327-AT-1 prostate cancer tumor showed accumulation of the tracer preferentially at the periphery rather than the center of the tumor, which could be attributed to the heterogeneity of GRPR expression in the different regions of the tumor or to limited accessibility of the drug to the center of the tumor. Furthermore, blood clearance renal excretion of the Sc-conjugate was reported to be fast [162].

Scandium radiotracers that are guided to their biological targets due to the presence of a particular organic fragment in their structure rather than an entire peptide or peptide-like targeting moiety have also been described. One example involved a 2-nitroimidazole-containing Sc-complex with a propensity for accumulation to hypoxic regions (entry 8, Table 5) [139]. The occurrence of hypoxia in the tumor microenvironment is known to promote cancer cell growth and angiogenesis, rendering hypoxic tumors a prime target for anticancer drug development. The reported ^44^Sc-DO3AM complex, equipped with 2-nitroimidazole (NI) (entry 5, Table 4), acted as a hypoxia probe, thus facilitating the mapping of hypoxic regions in tumors by means of PET imaging. The ability of the 2-nitroimidazole fragment to selectively mark hypoxic regions is due to its reduction and entrapment by nitroreductases, which are abundant in hypoxic cells. On the contrary, in normal cells, 2-nitroimidazole is re-oxidized and eliminated. The in vivo and ex vivo biodistribution of ^44^Sc-DO3AM-NI, in comparison to a ^68^Ga-counterpart, in both healthy and KB tumor-bearing SCID mice, were studied 90 and 240 min after intravenous administration. Both tracers accumulated with similar efficiency in KB tumors, but lower accumulation of the ^44^Sc-tracer compared to the ^68^Ga-tracer was observed in the liver, spleen, kidney, intestine, lung, heart, and brain. However, the ^44^Sc-tracer exhibited a 10- to 15-fold higher tumor-to-muscle ratio than the ^68^Ga-tracer at all time points. Therefore, ^44^Sc-DO3AM-NI was deemed a promising hypoxia probe.

Collectively, these pre-clinical studies, as well as the preliminary clinical data, suggest that DOTA and some of its structural analogues, labeled with Sc-44 and Sc-47, can form viable conjugates when paired to target-directing moieties, which, in turn, have great potential to be further developed as radiopharmaceuticals in diagnostic imaging and in nuclear medicine for the treatment of different types of cancer or other diseases. Despite the practical complexities, scandium radiopharmaceuticals appear to have similar biological effects to those of the widely explored Ga-68. Further studies on all aspects of scandium radiopharmaceutical development are required, especially for the diagnosis and treatment of cancer.

## 6. Future Prospects and Challenges in the Development of Scandium-DOTA Analogues

Many in vitro, in vivo, and preliminary clinical findings, which have been discussed in the previous section, support an increase in the efforts for further development of scandium-DOTA-type constructs/conjugates for clinical use as radiopharmaceuticals for the diagnosis and treatment of different diseases and in particular cancer.

The procedures for regulatory approval of diagnostic and theranostic radiopharmaceuticals are tedious and expensive. However, such procedures can be more “relaxed” for scandium radionuclide complexes with chelating agents such as DOTA and analogues, which can be used for cancer patient categories with no effective treatments at present, similar to other orphan drug development procedures [13,153].

Further clinical and other studies are required for the selection of the most appropriate scandium radiopharmaceutical for each different cancer category, including structure/activity correlation, toxicological, pharmacological, and cost evaluation studies, similar to other (chelating) drugs [162,163,164,165]. In all these cases, a risk/benefit assessment for the selection of drugs exhibiting optimal therapeutic activity, in the context of personalized medicine, will be required [165,166].

The interactions of DOTA and analogues, as well as their scandium complexes, with other metal ions and chelators including transferrin and lactoferrin are of great therapeutic, pharmacological, and toxicological importance [16,17,18,167,168]. In such cases, transmetallation and trans-chelation may cause changes in therapeutic outcomes, including toxic side effects. The same dilemmas apply to metabolites of DOTA and analogues. Furthermore, the toxicity and metabolic aspects of scandium radionuclides, following administration of related radiopharmaceuticals, must be investigated for short- and long-term toxic side effects, similar to gallium radionuclides following administration of gallium radiopharmaceuticals [169,170,171,172]. In challenging cases, the administration of deferiprone or other chelating drugs may be needed for the rapid elimination of scandium and gallium radionuclides following PET and SPECT scans in order to prevent scandium or gallium radionuclide associated toxicity [173,174].

Several other approaches may be considered for the development and utilization of DOTA/DOTA analogues-based scandium radiopharmaceuticals in cancer therapy and diagnosis, including combination with other radiopharmaceuticals or other anticancer drugs or procedures, such as photodynamic therapy, which is also used in certain types of cancer [175].

Further research on the design and development of new specific polyamino-polycarboxylates, alpha-hydroxypyridones, and other more specific chelators for scandium radionuclides, which can overcome existing complications and contribute in the optimization of diagnostic and therapeutic procedures, may be needed.

The future of the types of scandium radiopharmaceuticals discussed in this review for the treatment of cancer and potentially other diseases looks promising, especially since there are currently no effective therapies for many categories of cancer. In particular, the targeting of metastasis and addressing the challenges imposed by anticancer drug resistance encountered for many cancer patients are two areas of high priority, for which scandium radiopharmaceuticals have a potential to contribute on a therapeutic level [176,177,178,179].

## 7. Conclusions

Radiopharmaceuticals constitute a rapidly expanding category of pharmaceuticals, especially developed for diagnostic and theranostic applications in nuclear medicine, most importantly in the treatment of cancer. Scandium radioisotopes and scandium chelators appropriate for clinical application define an emerging area of interest in modern medicine that is closely connected to the design of sophisticated, high-precision equipment such as PET and SPECT scanners.

A pivotal aspect of this evolutionary trajectory in radiopharmaceuticals is the exploration and utilization of scandium isotopes Sc-43, Sc-44, and Sc-47, heralded as promising candidates for diagnostic and theranostic radiopharmaceuticals. However, their production necessitates multi-faceted methodologies involving nuclear reactors, cyclotron facilities, and radionuclide generators. While various nuclear reactions contribute to the production of these isotopes, challenges persist in achieving optimal yields and purity.

Concerning radiolabeling, the coordination of scandium ions with the DOTA chelator has led to the successful preparation of complexes exhibiting high stability, albeit with limitations such as slow formation kinetics and low labeling efficiency at ambient temperatures. Novel chelators like AAZTA have surfaced to address these shortcomings, showcasing enhanced kinetics and labeling efficiency across a range of temperatures. Nevertheless, disparities in stability and bio-distribution patterns observed with different analogues underscore the need for further investigation into optimal chelation strategies.

The clinical application of scandium radiopharmaceuticals demands rigorous preclinical and clinical evaluations encompassing pharmacological, toxicological, and efficacy studies. Furthermore, the potential synergies between scandium radiopharmaceuticals and other anticancer agents warrant exploration, offering avenues for enhanced therapeutic efficacy, as well as patient outcomes. Critical evaluation and refinement of these emerging technologies are imperative to realize their full potential in clinical practice.

## Figures and Tables

**Table 1:** Reported nuclear reactions for the production of Sc-43 and Sc-44, and the sources used.

Isotope	Nuclear Reaction	Target Structure	References
Sc-43	^43^Ca(p,n)^43^Sc	^nat^CaCO_3_, ^43^CaCO_3_	[43,62,79]
	^44^Ca(p,2n)^43^Sc	^44^CaCO_3_, CaCO_3_, CaO	[43,87]
	^46^Ti(p,α)^43^Sc	^46^TiO_2_	[61,74,87]
	^42^Ca(d,n)^43^Sc	^42^Ca, ^42^CaCO_3_	[74,82,87]
	^nat^Ca(α,n)^43^Ti (t_1/2_ = 509 ms)➔^43^Sc	^40^Ca, ^nat^CaCO_3_, ^40^CaCO_3_	[76,80,82]
	^nat^Ca(α,p)^43^Sc	^nat^CaCO_3_, ^40^CaCO_3_	[77,80,82]
Sc-44	^45^Sc(p,2n)^44^Ti (t_1/2_ = 60 y) (generator ^44^Ti➔^44^Sc)	^45^Sc	[3,69,70,85,88]
	^43^Ca(d,n)^44g^Sc	CaCO_3_, CaO	[82]
	^nat^Ca(p,n)^44^Sc	^nat^Ca(NO_3_)_2_	[29]
	^44^Ca(p,n)^44^Sc/^44m^Sc	^44^CaCO_3_, CaCO_3_, CaO	[29,43,82]
	^44^Ca(p,n)^44^Sc/^44m^Sc	^44^CaO	[77,83]
	^44^Ca(d,2n)^44^Sc/^44m^Sc	^44^CaCO_3_	[78,86]
	^44^Ca(a,n)^44^Sc	^44^CaO	[81]
	^47^Ti (p,α)^44^Sc	^47^TiO_2_	[75]

Abbreviations: d: deuteron, g: ground state, m: metastable state, n: neutron, nat: natural element, p: proton, ms: milliseconds, t_1/2_: half-life, α: alpha particle. (Note: References are numbered as they appear in the main text).

**Table 2:** Reported nuclear reactions for the production of Sc-47, and the sources used.

Isotope	Nuclear Reaction	Target Structure	References
Sc-47	^46^Ca(n,γ)^47^Ca → ^47^Sc	^46^CaCO_3_	[69,79,82,95,96,97,106]
	^nat^Ca(n,γ)^47^Ca → ^47^Sc	^nat^Ca	[90,105,106]
	^48^Ca(p,2n)^47^Sc	^48^CaCO_3_	[75]
	^44^Ca(α,p) ^47^Sc	^44^CaO	[59,81]
	^nat^Ti(n,p)^47^Sc	^nat^Ti	[93,111]
	^47^Ti(n,p)^47^Sc	^47^TiO_2_	[80,81,90,91,112]
	^50^Ti(p,α)^47^Sc	^50^TiO_2_	[113]
	^48^Ti(p,2p)^47^Sc	^48^TiO_2_	[104,113]
	^50^Ti(d,αn)^47^Sc	^50^TiO_2_	[113]
	^49^Ti(d,α)^47^Sc	^49^TiO_2_	[113]
	^47^Ti(d,2p)^47^Sc	^47^TiO_2_	[113]
	^48^Ti(γ,p)^47^Sc	^48^TiO_2_	[113,114]
	^51^V(p,αp)^47^Sc	^nat^V	[43]
	^nat^V(p,αp)^47^Sc	^nat^V	[103]
	^nat^V(p,x)^47^Sc	^nat^V	[100,107,108,109]

Abbreviations: d: deuteron, nat: natural element, n: neutron, p: proton, x: alpha or beta particle, α: alpha particle, γ: gamma radiation. (Note: References are numbered as they appear in the main text).

**Table 3:** Methods developed for the separation of the scandium isotopes from the target material and the corresponding recovery yields.

Target Material	Separation Method	Recovery Yield (%)	References
CaO	Extraction and ion exchange resins	-	[49,90]
CaCO_3_	Extraction chromatography	80	[40,91]
		93	[33]
		75	[44]
		95	[51,59,92]
		52–79	[77]
		95	[93]
	Ion exchange column	77	[12,87,94]
	Sinking	-	[47,50]
Ti(0)	Extraction chromatography	88	[86,87]
ΤiO_2_	Ion exchange column	88	[97]
	Extraction chromatography	97.7	[97]

**Table 4:** Structures of representative chelating agents, modelled after DOTA, which are used for the radiolabeling of Sc-43, Sc-44, and Sc-47.

Entry	Chelator Structure	Acronym	References
**1**	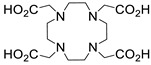	DOTA	[95,135,136]
**2**	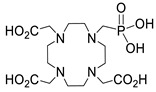	DO3AP	[137]
**3**	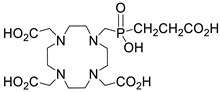	DO3AP^PrA^	[137]
**4**	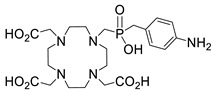	DO3AP^ABn^	[137]
**5**	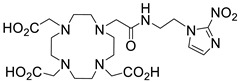	DO3AM-NI	[138]
**6**	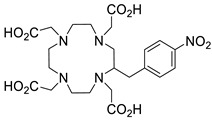	NBD	[139]
**7**	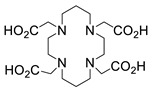	TETA	[135]
**8**	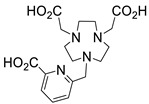	H_3_mpatcn	[141]
**9**	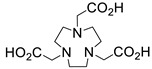	NOTA	[142,143]
**10**	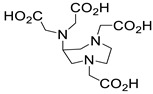	AAZTA	[142,144]
**11**	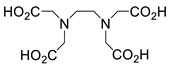	EDTA	[135]
**12**	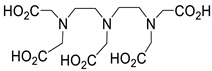	DTPA	[136]
**13**	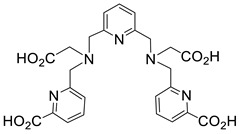	H_4_pypa	[145,146]

Note: The IUPAC names of all chelators included in this table can be found in the “Abbreviations” section.

## Abbreviations

A	Ampere
A (in chemical element symbol)	Mass Number
A7R	Ala-Thr-Trp-Leu-Pro-Pro-Arg
AAZTA	2,2-((1,4-Bis(carboxymethyl)-1,4-diazepan-6-yl)azanediyl)diacetic Acid
Ah	Ampere-hour
BN	Bombesin
Bq	Becquerel
cRGD	Cyclic Arginylglycylaspartic Acid
CT	Computed Tomography
D	Deuteron
DGA	Diglycolamide
DO3AM-NI	2,2′,2″-(10-(2-((2-(2-Nitro-1*H*-imidazol-1-yl)ethyl)amino)-2-oxoethyl)-1,4,7,10-tetraazacyclododecane-1,4,7-triyl)triacetic Acid
DO3AP	2,2′,2″-(10-(Phosphonomethyl)-1,4,7,10-tetraazacyclododecane-1,4,7-triyl)triacetic Acid
DO3AP^ABn^	2,2′,2″-(10-(((4-Aminobenzyl)(hydroxy)phosphoryl)methyl)-1,4,7,10-tetraazacyclododecane-1,4,7-triyl)triacetic Acid
DO3AP^PrA^	2,2′,2″-(10-(((2-Carboxyethyl)(hydroxy)phosphoryl)methyl)-1,4,7,10-tetraazacyclododecane-1,4,7-triyl)triacetic Acid
DOTA	2,2′,2″,2‴-(1,4,7,10-Tetraazacyclododecane-1,4,7,10-tetrayl)tetraacetic Acid
DOTA-NOC	2,2′,2″-(10-(2-((1-((13-((1*H*-Indol-3-yl)methyl)-10-(4-aminobutyl)-4-((1,3-dihydroxybutan-2-yl)carbamoyl)-7-(1-hydroxyethyl)-16-(naphthalen-1-ylmethyl)-6,9,12,15,18-pentaoxo-1,2-dithia-5,8,11,14,17-pentaazacycloicosan-19-yl)amino)-1-oxo-3-phenylpropan-2-yl)amino)-2-oxoethyl)-1,4,7,10-tetraazacyclododecane-1,4,7-triyl)triacetic Acid
DOTA-PSMA-617	((1-Carboxy-5-(3-(naphthalen-2-yl)-2-(4-((2-(4,7,10-tris(carboxymethyl)-1,4,7,10-tetraazacyclododecan-1-yl)acetamido)methyl)cyclohexane-1-carboxamido)propanamido)pentyl)carbamoyl)glutamic Acid
DOTA-RGD	2,2′,2″-(10-(2-((4-(14-Benzyl-11-(carboxymethyl)-5-(3-guanidinopropyl)-3,6,9,12,15-pentaoxo-1,4,7,10,13-pentaazacyclopentadecan-2-yl)butyl)amino)-2-oxoethyl)-1,4,7,10-tetraazacyclododecane-1,4,7-triyl)triacetic Acid
DOTATOC	2,2′,2″-(10-(2-((1-((13-((1*H*-Indol-3-yl)methyl)-10-(4-aminobutyl)-4-((1,3-dihydroxybutan-2-yl)carbamoyl)-16-(4-hydroxybenzyl)-7-(1-hydroxyethyl)-6,9,12,15,18-pentaoxo-1,2-dithia-5,8,11,14,17-pentaazacycloicosan-19-yl)amino)-1-oxo-3-phenylpropan-2-yl)amino)-2-oxoethyl)-1,4,7,10-tetraazacyclododecane-1,4,7-triyl)triacetic Acid
^D^R7A	DArg-DPro-DPro-DLeu-DTrp-DThr-DAla
DTPA	2,2′,2″,2‴-((((Carboxymethyl)azanediyl)bis(ethane-2,1-diyl))bis(azanetriyl))tetraacetic Acid
E	Energy of an Incident Photon in a Scintillator
e^−^	Electron
e^+^	Positron
EDTA	2,2′,2″,2‴-(Ethane-1,2-diylbis(azanetriyl))tetraacetic Acid
ELISA	Enzyme-Linked Immunosorbent Assay
eV	Electron Volt
FISRE	Free-Ion Selective Radiotracer Extraction
g	Ground State
GRPR	Gastrin Releasing Peptide Receptors
H_3_mpatcn	2,2′-(7-((6-Carboxypyridin-2-yl)methyl)-1,4,7-triazonane-1,4-diyl)diacetic Acid
H_3_NOTA	1,4,7-Triazacyclononane-1,4,7-triacetic Acid
H_4_pypa	6,6′-(((Pyridine-2,6-diylbis(methylene))bis((carboxymethyl)azanediyl))bis(methylene))dipicolinic Acid
HDEHP	Di-2-ethylhexyl Phosphoric Acid
HFIR	High Flux Isotope Reactor
HPGe	High-Purity Germanium
HPLC	High Performance Liquid Chromatography
ID/g	Injected Dose per Gram of Radionuclide
iTLC	Instant Thin-Layer Chromatography
K4R	Lys(HArg)-Dab-Pro-Arg
LNCaP	Human Prostate Cancer Cell Line
log K	Stability Constant
log K_i_	Protonation Constant
m	Metastable State
MBq/μmol	Megabecquerel per Micromole or Apparent Molar Activity
MCi	Millicurie
MP-AES	Microwave Plasma-Atomic Emission Spectrometry
MRI	Magnetic Resonance Imaging
N	Number of Neutrons
nat-V	Natural Vanadium
NBD	2,2′,2″,2‴-(2-(4-Nitrobenzyl)-1,4,7,10-tetraazacyclododecane-1,4,7,10-tetrayl)tetraacetic Acid
NI	2-Nitroimidazole
NODAGA-NOC	2,2′-(7-(4-((1-((13-((1*H*-Indol-3-yl)methyl)-10-(4-aminobutyl)-4-((1,3-dihydroxybutan-2-yl)carbamoyl)-7-(1-hydroxyethyl)-16-(naphthalen-1-ylmethyl)-6,9,12,15,18-pentaoxo-1,2-dithia-5,8,11,14,17-pentaazacycloicosan-19-yl)amino)-1-oxo-3-phenylpropan-2-yl)amino)-1-carboxy-4-oxobutyl)-1,4,7-triazonane-1,4-diyl)diacetic Acid
NODAGA-RGD	2,2-(7-(1-Carboxy-4-((4-(11-(carboxymethyl)-5-(3-guanidinopropyl)-14-(4-hydroxybenzyl)-3,6,9,12,15-pentaoxo-1,4,7,10,13-pentaazacyclopentadecan-2-yl)butyl)amino)-4-oxobutyl)-1,4,7-triazonane-1,4-diyl)diacetic Acid
NOTA	2,2′,2″-(1,4,7-Triazonane-1,4,7-triyl)triacetic Acid
NRP-1 protein	Neuropilin-1 Protein
p	Proton
PC-3 cells	Prostate Cancer cells
PET	Positron Emission Tomography
picagaDUPA	17-(4-(Carboxymethyl)-7-((6-carboxypyridin-2-yl)methyl)-1,4,7-triazonan-1-yl)-5,10,14-trioxo-4,6,11,13-tetraazaheptadecane-1,3,7,17-tetracarboxylic Acid
PSI	Paul Scherrer Institute
PSMA	Prostate-Specific Membrane Antigen
PTFE	Polytetrafluoroethylene
RGD	Arg-Gly-Asp or Arginylglycylaspartic Acid
s	Seconds
SCID	Severe Combined Immunodeficiency Disease
SCX	Strong Cation Exchange
SEM	Scanning Electron Microscopy
SiPM	Silicon Photomultiplier
SPECT	Single-Photon Emission Computed Tomography
SPION	Super-Paramagnetic Iron Oxide Nanoparticles
t_1/2_	Half-life
TBP	Tributyl Phosphate
TEM	Transmission Electron Microscopy
TETA	2,2′,2″,2‴-(1,4,8,11-Tetraazacyclotetradecane-1,4,8,11-tetrayl)tetraacetic Acid
TLC	Thin-Layer Chromatography
UTEVA	Uranium and Tetravalent Actinide
UV	Ultraviolet
V/M	Volume-to-Muscle ratio
VECC	Variable Energy Cyclotron Center
x	Alpha or Beta Particle
X	Position of an Incident Photon in a Scintillator on X-Axis
X (in nuclear decay reactions)	Parent Nucleus
X*	Excited Nuclear State of Parent Nucleus
Y	Position of an Incident Photon in a Scintillator on Y-Axis
Υ (in nuclear decay reactions)	Daughter Nucleus
y	Years
Z	Atomic Number
α	Alpha Particle
β	Beta Particle
β^+^	Positron
γ	Gamma Radiation
μL	Microliters
